# Travis County Medical Society

**Published:** 1888-11

**Authors:** B. F. Church

**Affiliations:** Secretary


### TRAVIS COUNTY MEDICAL SOCIETY.

Regular meeting held in Austin, October 4th, 1888, the President, John Preston, M. D., in the chair.

Dr. Q. C. Smith read a paper on "Antipyretics." A copy of the paper will be presented to the Society for their library.

DISCUSSION.

Dr. Bennett began the discussion by stating that while Dr. Smith's paper opened a wide field for study, he was somewhat disappointed that he did not enter into the details of the practical application of antipyretics. He thought heat reducing agents were not always indicated in fever. On the other hand, harm frequently resulted from their indiscriminate use. He had seen good results from the administration of Kairine, but cited a case in which rigors came on after giving it; was not certain, though, that this was a direct effect of the drug. Thought large doses of quinine dangerous.

Dr. Tyner agreed with Dr. Bennett that to reduce the temperature did not always cure the disease. There exists in all specific infectious diseases an alteration in the blood more or less profound, and we possess no specific for this condition. He believed in the theory that fever was an effort of nature to throw off the disturbing element and that antipyretics do harm in a great many instances by inhibiting this process.

Dr. Mathews, visiting from the city, said that he had seen, and was convinced that the most brilliant results in medicine were accomplished by the rational administration of antipyretics, and that they were indicated in all hyperpyrexiæ. He generally used antipyrine.

Dr. Lehde remarked, that he generally used quinine as an antipyretic, but had seen unfavorable symptoms from even moderate doses.

Dr. J. M. Litten thought that in the treatment of fevers we should pay more attention to the cause. He had had little experience with antipyrine. As a rule he gave quinine or the salicylates ; saw one case of syncope from a moderate dose of antipyrine. The salicylates should also be used with caution. Feed well and stimulate with brandy. He thought warm water better for reducing the temperature of typhoid fever than cold.

Dr. Hamilton, of Weberville, was of the opinion that pyrexia should always be reduced, and could not understand how fever could be a conservative force to throw off the poison from the system.

Dr. Tyner said, that Dr. Hamilton had doubtless conceived a wrong impression of his views ; he would not have antipyretics swept from existence, for they were often of inestimable value, and only condemned their injudicious use, especially in the specific fevers.

Dr. Bennett wishes also to not be misunderstood, and in substance agreed with Dr. Tyner.

The President, Dr. Preston, referred to two cases of typhoid fever, in which he kept the pyrexia in safe bounds with salicylate of soda. Under any condition when there is a high bodily temperature, food should not be forced upon the patient, as it could not be assimilated under such circumstances. In the use of antipyretics he was in favor of giving small doses, often repeated.

B. F. Church, M. D.,
Secretary.

